# Theoretical and Experimental Insights of Benzimidazole Catalyzed by the Epoxy–Acrylic Acid Reaction

**DOI:** 10.3390/molecules27227900

**Published:** 2022-11-15

**Authors:** Muhammad Jawwad Saif, Fazal-ur-Rehman, Shazia Abrar, Arruje Hameed, Nazeran Idrees, Muhammad Asif

**Affiliations:** 1 Department of Applied Chemistry, Government College University Faisalabad, Faisalabad 38000, Pakistan; 2 Department of Biochemistry, Government College University Faisalabad, Faisalabad 38000, Pakistan; 3 Department of Mathematics, Government College University Faisalabad, Faisalabad 38000, Pakistan

**Keywords:** reaction kinetics, esterification reaction, epoxy acrylate vinyl ester resin, computational study of epoxy acrylates

## Abstract

This study focuses on the experimental and molecular-level investigation of epoxy acrylate formation. Epoxy acrylate vinyl ester resin was prepared by a reaction of diglycidyl ether of bisphenol-A-based epoxy resin and acrylic acid, using benzimidazole as a catalyst. It was confirmed that benzimidazole can effectively catalyze this reaction. FTIR analysis of the product revealed a simple addition esterification reaction between the epoxide group and carboxylic group of acrylic acid excluding the side reactions (e.g., etherification). DFT computational studies were performed to theoretically explore the insights of reaction mechanisms. The calculations revealed that the benzimidazole-catalyzed reaction dominates the uncatalyzed reaction. A comparison of calculated activation energies showed that concerted mechanisms are less significant in such reactions owing to their high activation barriers.

## 1. Introduction

Vinyl ester resin (VER) is synthesized by the esterification reaction of an epoxy resin with an unsaturated monocarboxylic acid. VERs are important due to their vast applications in adhesive materials, printing inks, coatings, composites, and insulating materials [1,2,3,4]. Cured epoxy acrylate VERs display excellent electrical, thermal, and mechanical properties [5]. These resins are popular for their corrosion resistance [5], low moisture absorption [6], and thermal stability [7,8,9].

Traditionally, amines have been considered major catalysts for esterification reactions of epoxies. Many researchers have reported the kinetic studies of addition esterification reactions involving mono-epoxy and di-epoxy [10,11,12,13,14]. These studies involved different catalysts such as *N*,*N*-dimethyltoluidine, trihexylamine, quinolone, pyridine, dimethylbenzylamine, etc.

**Scheme 1:**
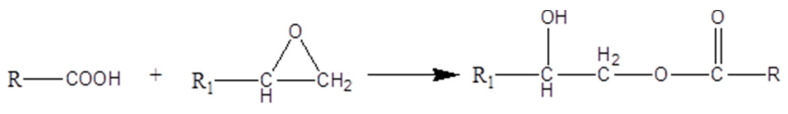
Addition esterification.

**Scheme 2:**
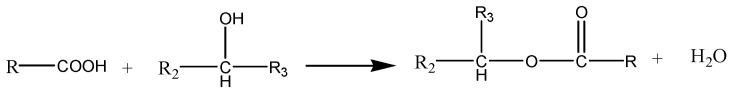
Condensation esterification.

**Scheme 3:**
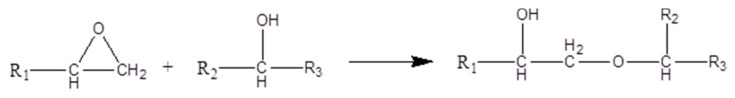
Etherification.

It is noted that three possible reactions can occur during acid and epoxy reactions which are given below (Scheme 1, Scheme 2 and Scheme 3):

(1) The reaction between the carboxylic group and the epoxide group.
(2) The reaction between carboxylic and –OH groups on epoxy resin chains.
(3) The reaction between epoxide and –OH groups.

In the course of an epoxy acid chemical reaction, the epoxide and acid values decrease, whereas the viscosity of epoxy acrylate vinyl ester resins increases. Unlike etherification, during condensation esterification and addition esterification reactions, there is a slight viscosity rise for a huge reduction in acid value. Moreover, there is a possibility of linking unsaturated acids with each other through a free radical mechanism. In extreme cases, it may cause gelation. The addition esterification reactions are generally proceeded under appropriate reaction conditions and using suitable catalysts. Gelation is controlled using an inhibitor [15].

The present study focuses on the working mechanism of benzimidazole (as a catalyst) and the kinetics of the reaction between acrylic acid and bisphenol-A-based epoxy resin for an addition esterification reaction.

First, we investigated the order of reaction by a differential method, and then it was confirmed by an integral method. Different mechanisms have been proposed in the literature for an epoxy-carboxylic acid reaction (see Discussion) [16].

With advancements in computational models, it is becoming increasingly popular to study reaction mechanisms at a molecular level as they could offer more logical interpretations. Since different reaction mechanisms are proposed in the literature for such reactions, it becomes more important and pertinent to computationally explore the mechanism insights.

## 2. Results and Discussion

**Figure 1:**
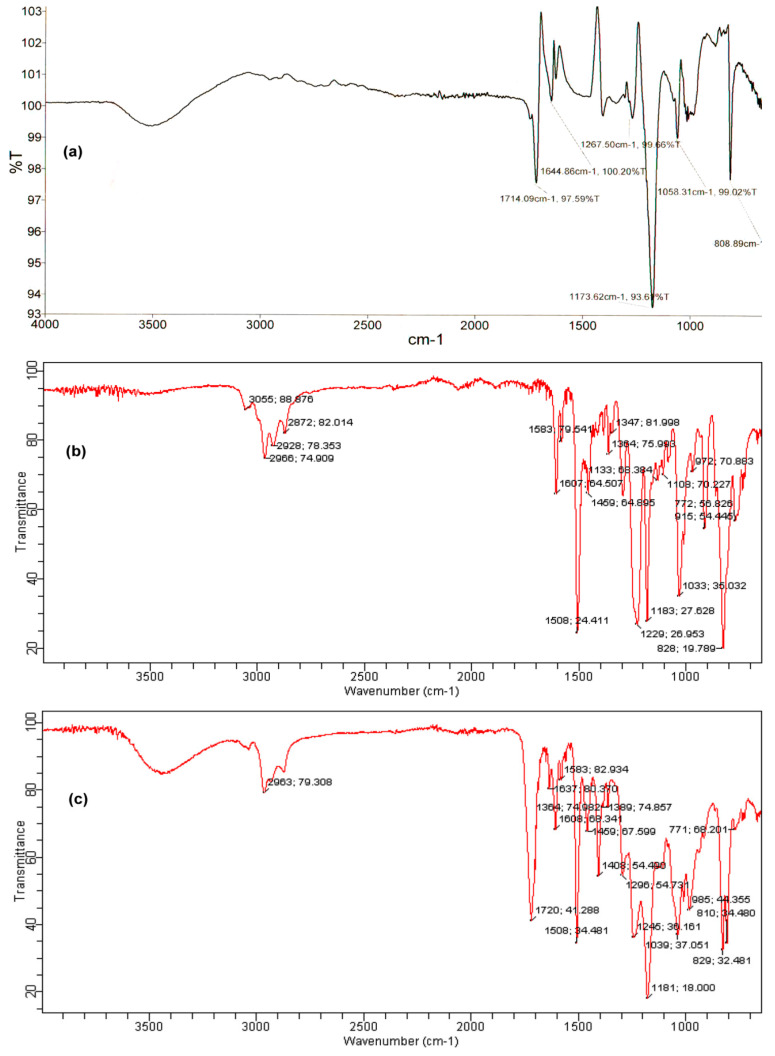
The FT-IR spectrum of (**a**) acrylic acid, (**b**) DGEBA epoxy resin, and (**c**) synthesized vinyl ester resin.

The FTIR spectra of acrylic acid, epoxy resin, and prepared epoxy acrylate vinyl ester resin are shown in Figure 1. The absorption peak at 915 cm^−1^ associated with the oxirane ring of epoxy resin is substituted by a band at 1720 cm^−1^ in the epoxy acrylate vinyl ester resin, which is referred to as the carbonyl group of ester. An additional band appears at 1637 cm^−1^ in epoxy acrylate vinyl ester spectra which refers to the acryloyl double bond, approving the preparation of epoxy acrylate vinyl ester resin. The absence of epoxide ring peak (915 cm^−1^) in epoxy acrylate vinyl ester resin reveals that epoxy groups reacted to form epoxy acrylate ester resin. Moreover, the absence of an ether absorption peak at 1120 cm^−1^ shows that the epoxy groups did not react with side-chain hydroxyl groups. The absence of an absorption peak at 1714 cm^−1^ (refers to a carboxylic group in acrylic acid in epoxy acrylate vinyl ester resin spectrum) also approves the accomplishment of acid-epoxy addition esterification reaction.

The esterification of epoxy acrylate was carried out in the presence of benzimidazole as a catalyst at three different temperatures (−80 °C, 90 °C, and 100 °C).

**Figure 2:**
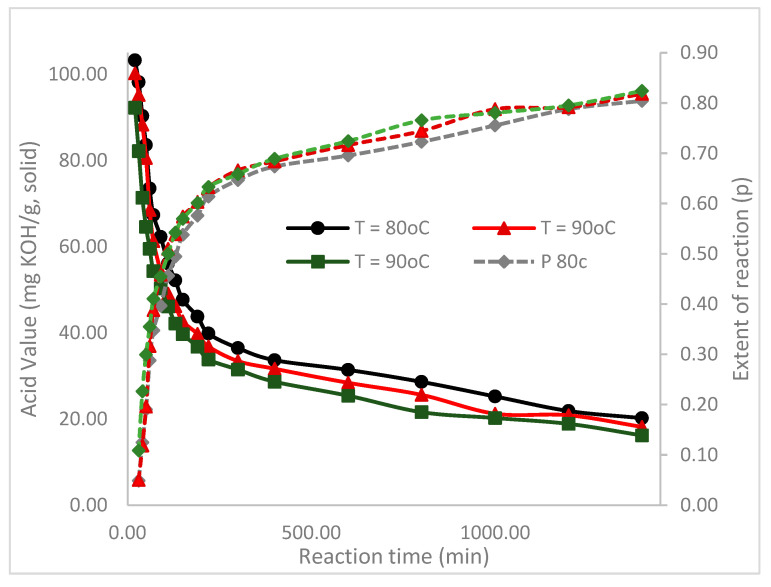
The acid value and extent of reaction (p) vs. reaction time (min).

Figure 2 shows the changes in acid values and the extent of reaction values for the time at different reaction temperatures. The profile resembles a typical esterification reaction. It is evident from the data that the acid value decreases with an increase in the reaction time and this decrease is linear at the initial steps of the reaction.

Values for the extent of reaction (p) and the number-average degree of polymerization (${\bar{\mathrm{DP}}}_{N}$) at 80 °C, 90 °C, and 100 °C are calculated using Carother’s equation [17] and the data are presented in Table 1. At all temperatures, ${\bar{\mathrm{DP}}}_{N}$ increases with the reaction times. However, this increase is non-linear at the initial stages of reaction (<40% conversion) and later stages (>75% conversions). This observation is characteristic of catalyzed esterification reactions.

We applied the first-order rate equation to the experimental data. First-order kinetics for epoxy-acrylic acid esterification has been suggested by many authors, though they have employed different catalysts [18]. The relationship for the first-order kinetic equation is:

(1) $$ln\frac{AV_{t}}{AV_{o}}=-K_{T}t$$

Rate constants were calculated using the least square method, expressed as:

(2) $$K_{T}=-\frac{\sum_{i=1}^{n}t_{i}\ln\left(\frac{AV_{t}}{AV_{o}}\right)}{\sum_{i=1}^{n}t_{i}^{2}}$$

**Figure 3:**
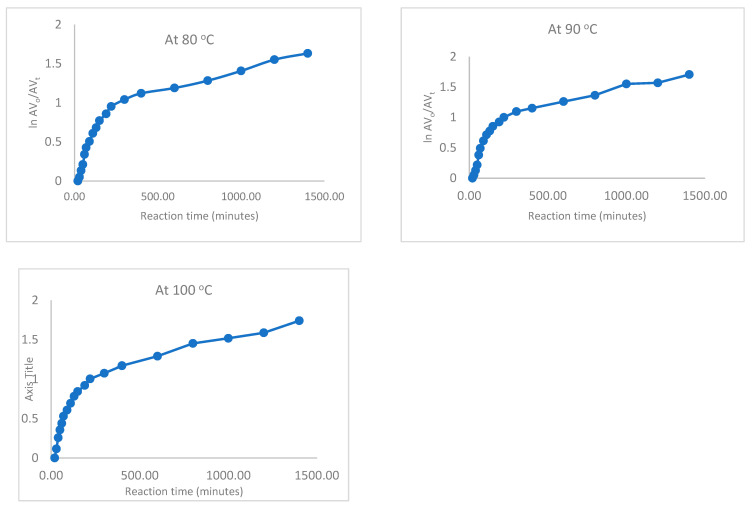
lnAV_o_/AV_t_ versus time at 80 °C, 90 °C, and 100 °C in the presence of benzimidazole as a catalyst.

Figure 3 presents the plot between lnAV_o_/AV_t_ and the reaction time at 80 °C, 90 °C, and 100 °C in the presence of benzimidazole as a catalyst. 

These plots represent data fitting for the first-order kinetic rate equation. Initially, it appears that the reaction efficiently follows the kinetic model in the first 400 min up to a 68% extent of reaction (Figure 3 and Table 1).

The rate appears to be more like the pseudo first order at the start, and then decays with change in the concentration of nucleophile as the reaction proceeds. This seems to be mitigated by the addition of the catalyst. This indicates that a single rate equation may not adequately represent the kinetics of the reaction. Rate constants and regression constants are provided in Table 2.

It should be particularly noted that several factors, including the reagent chemical structure, the type of catalyst, and solvent media, can influence the final observations. Although there is abundant literature available on non-catalyzed epoxy–acid reactions, basic (amine-based) catalysts have been popular as the former is very slow and of little interest in applications. Due to the complex reaction mechanism, it was hard to unambiguously interpret the reaction mechanisms.

There has been disagreement among researchers on the epoxy–acid crosslinking mechanism. A group of workers believes that epoxy and acid could react without an added catalyst. They are backed by the hypothesis that curing can auto-initiate by hydrogen donation with an acid or hydroxyl group or that curing can happen from an epoxy-acid complex. 

For catalyzed reactions, two pathways have been proposed. The first pathway proposes the formation of an epoxy–catalyst intermediate that further reacts with a carboxylic acid. The second pathway, which is generally more accepted, proposes the formation of a carboxylic acid–catalyst complex that further reacts with the epoxy. The latter pathway seems to be intuitionally more acceptable as amine and carboxylic acid could react together to form a salt.

There are good reviews available in the literature citing kinetic studies of epoxy–carboxylic acid reaction using various catalysts [16]. However, these kinetic models failed to interpolate the epoxy–carboxylic acid esterification reaction in general. DSC kinetic methods are also employed to study such esterification. One of the major drawbacks of the DSC method in such systems is that DSC cannot distinguish between α or β esters. 

**Scheme 4:**
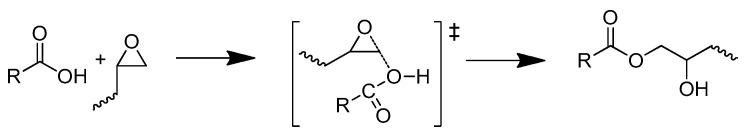
The epoxy–carboxylic acid reaction in the absence of catalyst (R_i_).

**Scheme 5:**
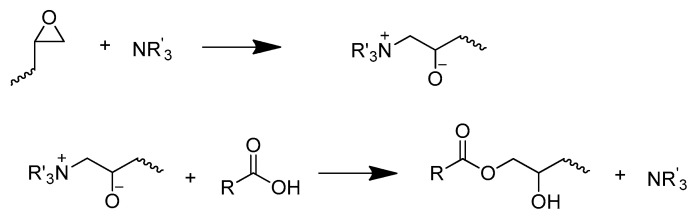
The epoxy–amine zwitterion pathway (Z_i_).

**Scheme 6:**
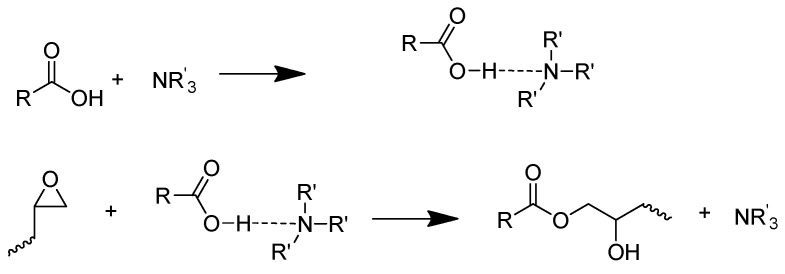
The acid–amine complex pathway (Raa).

The main objective of this study was to reinvestigate the experimental data and a thorough study of epoxy–carboxylic acid reactions at a molecular level. For this purpose, we made three suppositions based on the previous literature review [19]. First, epoxy–carboxylic acid can react in the absence of a catalyst [20] (denoted by R_i_); second, the reaction pathway proceeds via epoxy–amine zwitterion (denoted by Z_i_), as proposed by Mares et al. [21]; and third, the acid–amine complex transforms the epoxy into the final product [22] (denoted by R_aa_). These pathways are illustrated in Scheme 4, Scheme 5 and Scheme 6.

In the literature, non-catalyzed reactions have been shown to form both primary and secondary esters. In the case of a base-catalyzed reaction, however, only primary esters have been reported earlier [23]. For the sake of consistency, we chose the primary ester formation for our computational studies.

**Figure 4:**
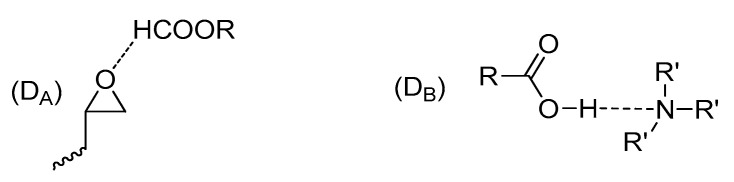
(D_A_) d(OH-O) = 1.806 Å, ΔE_bind_ = −32 kJmol^−1^, (D_B_) d(OH-N) = 1.711 Å, ΔE_bind_ = −51 kJmol^−1^.

The formation of a precursor complex between the carboxylic acid group, the catalyst, and the epoxy group is possible. Figure 4 represents such precursor complexes along with their respective binding energies, as calculated by computational methods (ΔE_bind_). 

**Scheme 7:**
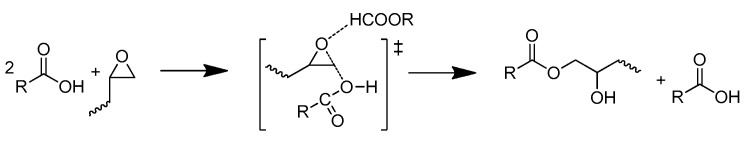
The hydrogen-bonded epoxy–carboxylic acid reaction in the absence of the catalyst (Ri-hb).

**Scheme 8:**
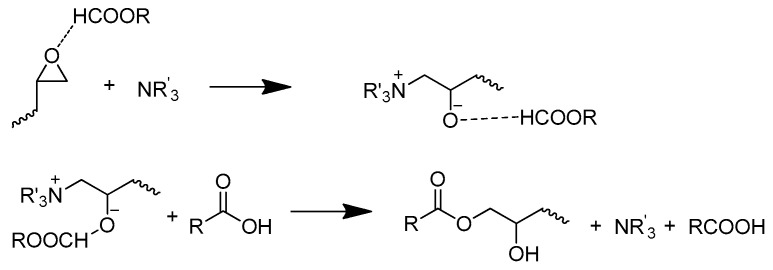
The hydrogen-bonded epoxy–amine zwitterion pathway (Zi-hb).

**Scheme 9:**
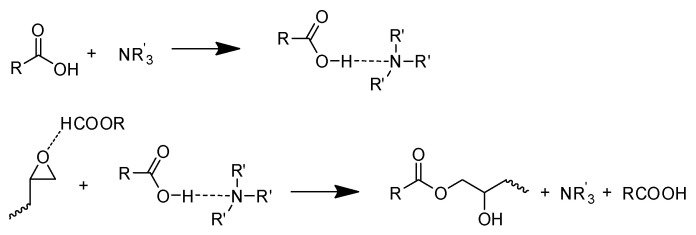
The hydrogen-bonded acid–amine complex pathway (Raa-hb).

Complexes D_A_ and D_B_ show possible interaction between the reactants. The binding energy of N…OH is greater as nitrogen is a better hydrogen acceptor than oxygen. This way, species D_A_ and D_B_ can also participate in the reaction along with epoxy, carboxylic acid, and the catalyst, as presented in Scheme 7, Scheme 8 and Scheme 9.

Computed classical energy barriers for transition states and products via different reaction pathways are presented in Table 3, and the transition states involved in proposed mechanisms are presented in Table 4.

The pathway Ri proceeds via a six-membered ring transition state where acidic OH groups bind with the oxygen of the epoxy group, whereas the carbonyl group attacks the methylene group of epoxy. This mechanism has been confirmed experimentally by the second-order reaction. An experimental re-investigation of the reaction revealed that ring opening is trans which is contradictory to this mechanism [24]. Computationally, it is found that a proton transfer from the hydroxyl group to epoxide oxygen is the rate-determining step in the Ri mechanism, unlike the other mechanisms where ring opening is the rate-determining step.

It has been experimentally established that the order of reaction dominantly depends on the reactants—the acidity of acid, the basicity of oxide, and the acid–oxide ratio. The Ri-hb pathway involves a hydrogen-bonded precursor complex (D_A_) that further reacts with an acidic group. The transition state involves a trans bonding position. The transition state has a lower energy barrier as compared to the Ri mechanism. This mechanism agrees well with literature reports describing a trans opening of the epoxide group.

The second proposed mechanism involves the opening of an epoxide ring by a catalyst, leading to the formation of a zwitterion. The zwitterion pathways Zi and Zi-hb, both proceed via two steps—the formation of zwitterion and subsequent curing. Transition states for step one is screened for energy barriers that led to the dominant pathway being chosen. It is observed that the energy barrier for the transition state in Rz-hb is lower than that of Rz, indicating that hydrogen bonding between the epoxide group and acidic -OH effectively lowered the activation energy for zwitterion formation. The epoxide ring opening was associated with a transfer of electron pairs from catalyst nitrogen to epoxide carbon. In the curing step, hydrogen transfer takes place to the epoxide oxygen and the required energy for such transfer can be considered negligible as compared to the energy barrier for zwitterion formation.

The pathways involving the acid–amine complex (R_aa_ and Raa-hb) also proceed via two steps. In the first step, there is an association between the acid and the catalyst. The second step is the curing step. The association step is fast as compared to the second one; hence, only the second step is focused. Intuitively, it can be expected that hydrogen bond formation between the catalyst and carboxylic acid increases the nucleophilicity of the carboxylic group. The computational results also reported a low energy barrier for the involved transition state in the Raa-hb reaction path. It is further observed that hydrogen-bonded precursors (DA and DB) involve lower energy consumption.

**Figure 5:**
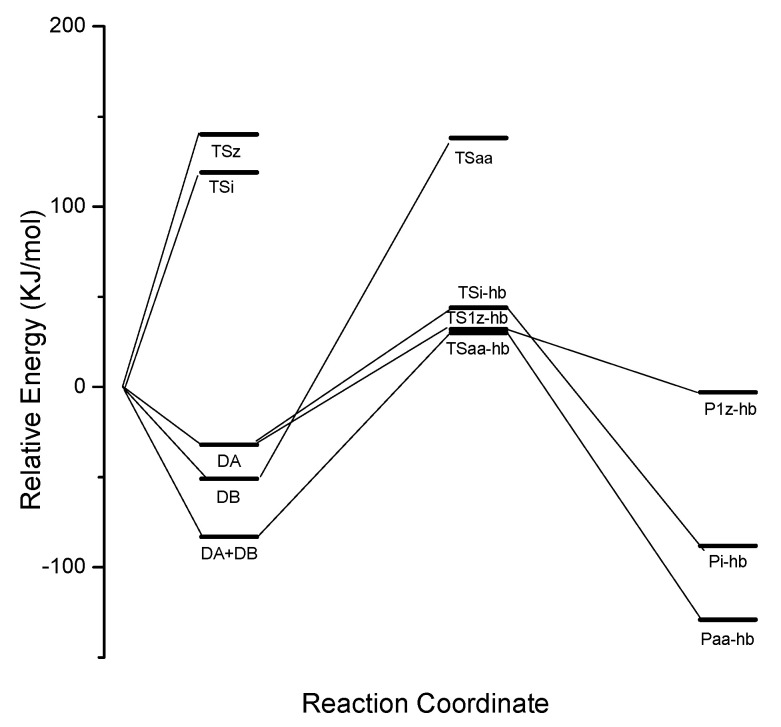
Potential energy diagram for the catalyzed epoxy-acrylic acid system.

Overall, the Raa-hb reaction path was found to be associated with the lowest energy barriers and the smallest heat consumption. This finding is in agreement with previous studies [25]. The potential energy diagram for various mechanisms is shown in Figure 5.

## 3. Experimental

### 3.1. Materials

Araldite F liquid, solvent-free and unmodified bisphenol-A epoxy resin (with a viscosity range of 9000–12,000 mPasm, and epoxide with an equivalent weight of 190) was purchased from the Huntsman group. Its epoxy content was 5.20–5.35 Equiv/kg (ISO 3001) with a density range of 1.15–1.20 g/cm^3^. Acrylic acid (AA) was supplied by power chemical industries (PVT) LTD, in Pakistan. Catalyst benzimidazole (lot no. 10141334) was purchased from Alfa Aesar.

### 3.2. Method

The benzimidazole catalyst (1 wt%) was added to stoichiometric amounts of acrylic acid and Araldite F. The esterification reaction was carried out at 80 °C, 90 °C, and 100 °C in separate experiments.

The progress of the reaction was monitored by acid values using a method by Ogg et al. [26]. Equation (3) relates the acid number of the reaction mixture to the acid group concentration.

(3) $$\mathrm{AV}=\frac{\left[\mathrm{Acid}\right]\times 56.1}{d}$$

The extent of the reaction was calculated from the acid values using the formula given below.

(4) $$P=1-\frac{M}{M_{0}}$$

*M* is the acid value of resin at the time ‘t’ and *M*_0_ is the acid value at time zero.

The kinetics of the reaction were studied using the power law model. The reaction order was found by the differential method. The energy of activation ($E_{a}$) and frequency factor (A) were calculated using the Arrhenius equation.

(5) $$K_{T}=Ze^{-E_{a}/RT}$$

The enthalpy of reaction (ΔH) and the free energy of activation (ΔG) were calculated using Expression (3).

(6) $$\Delta H=E_{a}-RT$$

Whereas, the entropy of the reaction was calculated using Expression (4).

(7) $$K_{T}=\frac{k_{b}T}{h}e^{-E_{a}/RT}e^{\Delta S/R}$$

### 3.3. Computational Models

In the present work, methyl glycidyl ether was chosen as a representative model of DGEBA, and imidazole was chosen for benzimidazole. Small representative molecules offer faster computation. All calculations were made using the Gaussian 09W program package. A hybrid density functional theory B3LYP with a 6-31G(d,p) basis set was employed to locate stationary points of reactants, transition states, and products. All stationary points were confirmed by vibrational frequency analysis. Transition states were confirmed by minimum energy reaction pathway (MERP) using the Gonzalez–Schlegel method in mass-weighted Cartesian coordinates from transition state to both reactants and products. The geometries of the transition states with zero-point energy corrections were used for comparison purposes. The calculated activation energies were thus compared with the experimental values.

## 4. Conclusions

Based on the study, the following conclusions can be made: (a) benzimidazole can effectively catalyze the epoxy–carboxylic acid reaction; (b) the order of reaction depends on the nature of reactants and experimental conditions; and (c) a computational study reveals that reaction proceeds via a complex formation between carboxylic acid and amine (catalyst).

## Figures and Tables

**Table 1:** The extent of reaction (p) and the degree of polymerization ${\bar{\mathrm{DP}}}_{N}$ vs. time (min).

Time (min)	80 °C		90 °C		100 °C	
	p	${\bar{DP}}_{N}$	p	${\bar{DP}}_{N}$	p	${\bar{DP}}_{N}$
30.00	0.05	1.05	0.05	1.05	0.11	1.12
40.00	0.12	1.14	0.12	1.13	0.23	1.29
50.00	0.19	1.23	0.20	1.24	0.30	1.43
60.00	0.29	1.40	0.32	1.46	0.35	1.55
70.00	0.35	1.53	0.39	1.63	0.41	1.70
90.00	0.40	1.66	0.46	1.85	0.45	1.83
110.00	0.46	1.84	0.51	2.04	0.50	2.00
130.00	0.49	1.98	0.54	2.17	0.54	2.19
150.00	0.54	2.16	0.57	2.35	0.57	2.32
190.00	0.58	2.36	0.60	2.52	0.60	2.51
220.00	0.61	2.59	0.63	2.72	0.63	2.73
300	0.65	2.83	0.67	2.99	0.66	2.93
400	0.67	3.07	0.68	3.17	0.69	3.22
600	0.70	3.29	0.72	3.53	0.72	3.63
800	0.72	3.61	0.74	3.91	0.77	4.27
1000	0.76	4.09	0.79	4.72	0.78	4.56
1200	0.79	4.72	0.79	4.80	0.80	4.88
1400	0.80	5.11	0.82	5.51	0.82	5.69

**Table 2:** Rate constants and regression constants.

T	First Order Rate Equation (6)	
	K	R
80 °C	0.0015	0.778
90 °C	0.0016	0.770
100 °C	0.00162	0.813

**Table 3:** Computed classical energy barriers for transition states and products via different reaction pathways at 0 K.

Pathway	Compound	ΔE (kJ/mol)
Ri	TSi	119
Ri-hb	TSi-hb	76
	Pi-hb	−46
Zi	TSzi	140
	Pzi	117
Zi-hb	TS1zi-hb	64
	P1zi-hb	29
Raa	TSaa	189
	Paa	−49
Raa-hb	TSaa-hb	113
	Paa-hb	−46

**Table 4:** Transition states involved in proposed mechanisms.

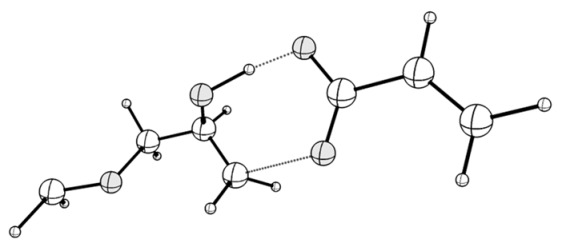	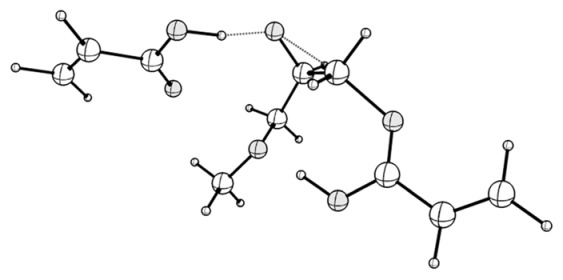
TS_i: <O1-C1-C2 = 88.42, C2-O2 = 2.286, ν (i, cm^−1^) = −771	TS_i_hb: <O1-C1-C2 = 91.84, C2-O2 = 1.757, ν (i, cm^−1^) = −402
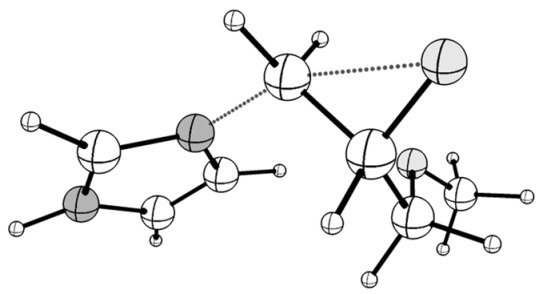	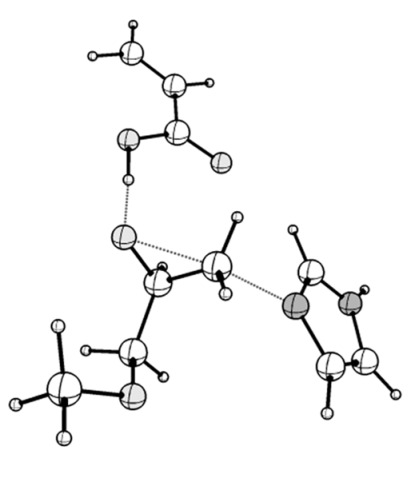
TS_1z: <O1-C1-C2 = 91.69, C2-N1 = 1.799, ν (i, cm^−1^) = −442	TS_1z_hb: <O1-C1-C2 = 82.87, C2-N1 = 2.030, ν (i, cm^−1^) = −456
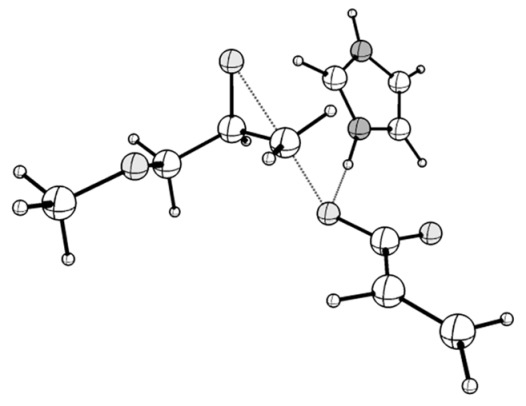	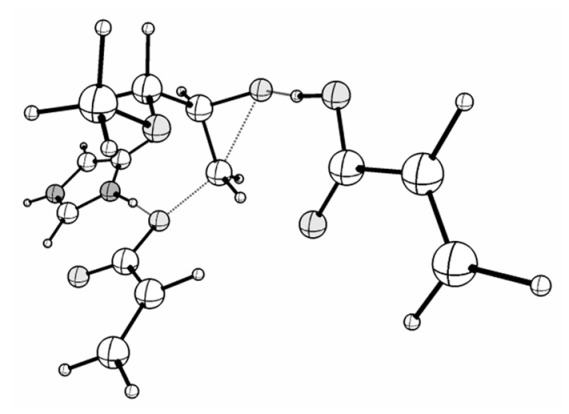
TS_Raa: <O1-C1-C2 = 90.38, C2-O2 = 1.899, ν (i, cm^−1^) = −449	TS_Raa-hb: <O1-C1-C2 = 89.53, C2-O2 = 1.837, ν (i, cm^−1^) = −482

## Data Availability

Not applicable.

## References

[1] Clay J., Armstrong S., Leotsakos G. (2019). High Performance Water-Based Adhesion Compositions and Applications. U.S. Patent.

[2] Olson D., Lin W., Seeger G. (2016). Adhesive Formulations for Bonding Composite Materials. U.S. Patent.

[3] Jaswal S., Gaur B. (2014). New Trends in Vinyl Ester Resins. Rev. Chem. Eng..

[4] Alía C., Jofre-Reche J., Suárez J. (2015). Influence of Post-Curing Temperature on the Structure, Properties, and Adhesion of Vinyl Ester Adhesive. J. Adhes..

[5] Taillemite S., Pauer R. (2009). Bright Future for Vinyl Ester Resins in Corrosion Applications. Reinf. Plast..

[6] Alateyah A.I., Dhakal H.N., Zhang Z.Y., Materials A. (2013). Water Absorption Behavior, Mechanical and Thermal Properties of Vinyl Ester Matrix Nanocomposites Based on Layered Silicate. Polym. Technol..

[7] Guo Z., Shin K., Karki A.B., Young D.P., Kaner R.B., Hahn H.T. (2009). Fabrication and Characterization of Iron Oxide Nanoparticles Filled Polypyrrole Nanocomposites. J. Nanoparticle Res..

[8] Guo Z., Liang X., Pereira T., Scaffaro R., Thomas Hahn H. (2007). CuO Nanoparticle Filled Vinyl-Ester Resin Nanocomposites: Fabrication, Characterization and Property Analysis. Compos. Sci. Technol..

[9] Alhuthali A., Low I.M., Dong C. (2012). Characterisation of the Water Absorption, Mechanical and Thermal Properties of Recycled Cellulose Fibre Reinforced Vinyl-Ester Eco-Nanocomposites. Compos. Part B Eng..

[10] Gawdzik B., Matynia T. (2001). Synthesis and Modification of Epoxy-based Divinyl Ester Resin. J. Appl. Polym. Sci..

[11] Firdous H., Bajpai M. (2010). UV Curable Heat Resistant Epoxy Acrylate Coatings. Chem. Chem. Technol..

[12] Srivastava A., Agrawal S., Rai J.S.P. (2002). Kinetics of Esterification of Cycloaliphatic Epoxies with Methacrylic Acid. J. Appl. Polym. Sci..

[13] Pal N., Srivastava A., Rai J.S.P. (2003). Synthesis of Vinyl Ester Resins in the Presence of Monoepoxies: A Kinetic Study. Polym. Plast. Technol. Eng..

[14] Kakiuchi H., Tanaka Y. (1966). Study of Epoxy Compounds. VII. 1,2 Base-Catalyzed Reaction of Substituted Phenyl Glycidyl Ethers with Benzoic Acid. J. Org. Chem..

[15] Oldring P.K.T., Hayward G. (1987). A Manual for Resin for Surface Coatings. Sita Technol.

[16] Madec P.-J., Maréchal E. (1985). Kinetics and Mechanisms of Polyesterifications II. Reactions of Diacids with Diepoxides. Analysis/Reactions/Morphology.

[17] Odian G. (2004). Principles of Polymerization.

[18] Srivastava A., Pal N., Agarwal S., Rai J.S.P. (2005). Kinetics and Mechanism of Esterification of Epoxy Resin with Methacrylic Acid in the Presence of Tertiary Amines. Adv. Polym. Technol..

[19] Sultania M., Rai J.S.P., Srivastava D. (2010). Kinetic Modeling of Esterification of Cardanol-Based Epoxy Resin in the Presence of Triphenylphosphine for Producing Vinyl Ester Resin: Mechanistic Rate Equation. J. Appl. Polym. Sci..

[20] Lebedev M., Guskov K. (1964). The Reactions of A-Oxides. IV. Investigation of Acid Catalyst and Intermediate Products during Reaction of Ethylene Oxide with Carboxylic Acids. Kinet. Katal.

[21] Mareš F., Hetflejš J., Bažant V. (1969). Reactions of Carboxylic Acids with Ethylene Oxide. I. Kinetics of the Reaction of Terephthalic Acid with Ethylene Oxide. Collect. Czechoslov..

[22] Samsonova T.I., Mikhailov G.D., Malykh V.A., Chegolya A.S. (1977). Production of Bis(β-Ethoxy)Terephthalate from Terephthalic Acid and Ethylene Oxide. Fibre Chem..

[23] Blank W.J., He Z.A., Picci M. (2002). Catalysis of the Epoxy-Carboxyl Reaction. J. Coat. Technol..

[24] Shvets V.F., Lebedev N.N., Tyukova O.A. (1971). Kinetics and Stereochemistry of Alph Epoxide Reactions with Alcohols and Carboxylic Acids without Catalysts. Zhurnal Org. Khimii.

[25] Sorokin M.D., Gershanov E.L. (1967). Kinetics and Mechanisms of Polyesterifications II. Reactions of Diacids with Diepoxides. Kinet. Katal..

[26] Ogg C.L., Porter W.L., Willits C.O. (1945). Determining the Hydroxyl Content of Certain Organic Compounds Macro-and Semimicromethods. Ind. Eng. Chem. Anal. Ed..

